# Enterocin Cross-Resistance Mediated by ABC Transport Systems

**DOI:** 10.3390/microorganisms9071411

**Published:** 2021-06-30

**Authors:** Claudia Teso-Pérez, Manuel Martínez-Bueno, Juan Manuel Peralta-Sánchez, Eva Valdivia, Mercedes Maqueda, M. Esther Fárez-Vidal, Antonio M. Martín-Platero

**Affiliations:** 1 Departamento de Microbiología, Universidad de Granada, Avda. Fuentenueva s/n, 18071 Granada, Spain; mmartine@ugr.es (M.M.-B.); jmps@ugr.es (J.M.P.-S.); evavm@ugr.es (E.V.); mmaqueda@ugr.es (M.M.); 2 Departamento de Bioquímica y Biología Molecular III e Inmunología, Facultad de Medicina, Universidad de Granada, 18016 Granada, Spain; 3 Instituto de Investigación Biomédica IBS, Granada, Complejo Hospitalario Universitario de Granada, Universidad de Granada, 18071 Granada, Spain

**Keywords:** enterocin, ABC transporter, bacteriocin immunity, MR10A/B, AS-48

## Abstract

In their struggle for life, bacteria frequently produce antagonistic substances against competitors. Antimicrobial peptides produced by bacteria (known as bacteriocins) are active against other bacteria, but harmless to their producer due to an associated immunity gene that prevents self-inhibition. However, knowledge of cross-resistance between different types of bacteriocin producer remains very limited. The immune function of certain bacteriocins produced by the *Enterococcus* genus (known as enterocins) is mediated by an ABC transporter. This is the case for enterocin AS-48, a gene cluster that includes two ABC transporter-like systems (Transporter-1 and 2) and an immunity protein. Transporter-2 in this cluster shows a high similarity to the ABC transporter-like system in MR10A and MR10B enterocin gene clusters. The aim of our study was to determine the possible role of this ABC transporter in cross-resistance between these two different types of enterocin. To this end, we designed different mutants (Tn*5* derivative and deletion mutants) of the *as-48* gene cluster in *Enterococcus faecalis* and cloned them into the pAM401 shuttle vector. Antimicrobial activity assays showed that enterocin AS-48 Transporter-2 is responsible for cross-resistance between AS-48 and MR10A/B enterocin producers and allowed identification of the MR10A/B immunity gene system. These findings open the way to the investigation of resistance beyond homologous bacteriocins.

## 1. Introduction

Most bacteria live within complex microbial communities in which they must compete for biotic and abiotic resources to ensure their survival. This competition can be passive, when one strain harms another through resource consumption, or active, when strains damage each other through chemical warfare (antagonistic interaction) [1]. An important antagonistic mechanism in bacteria involves the production of antimicrobial peptides (bacteriocins), a widely observed phenomenon [2]. Bacteriocin production has been observed in cyanobacteria [3], enterobacteria [4], and lactic acid bacteria (LAB) [5], among many other prokaryotes and *Archaea*. Bacteriocin production allows bacteria to improve the stability of their communities by competing against closely related bacterial species to establish a stable niche for the producer strain [6].

Bacteriocins are ribosomally synthesized antimicrobial peptides [7,8] that can have a broad spectrum of activity, as in the case of bacteriocins produced by Gram-positive bacteria such as bifidocin A [9], lacticin 3147 [10], and nisin [11]. Bacteriocins are a heterogeneous group of peptides that vary in size, structure, mode of action, and/or target cell receptor [2]. These differences give rise to three classes of bacteriocins: class I, small (less than 10KDa) heat-stable peptides that undergo post-translational modifications; class II, small (less than 10KDa) heat-stable, non-modified peptides; and class III, large thermolabile peptides [12,13].

**Figure 1:**
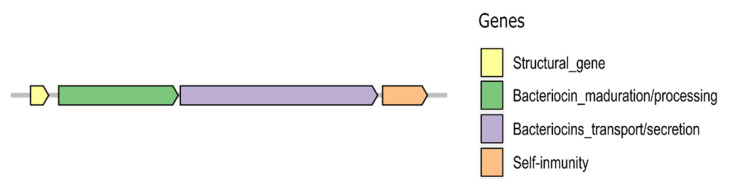
Common bacteriocin gene cluster organization. The general genetic organization for bacteriocin production involves four main types of genes: structural genes; genes involved in maturation, processing; genes involved in the transport, secretion of bacteriocin; and genes that confer self-immunity to the bacteriocin.

Bacteriocin production involves the coordinated expression of several groups of genes distributed in one or more operons located either on the chromosome or on plasmids. Producers must carry their own immunity gene in the gene cluster to avoid self-inhibition. Bacteriocin gene clusters usually include genes encoding (i) the bacteriocin structure; (ii) bacteriocin maturation and processing; (iii) bacteriocin transport and/or secretion; and (iv) self-immunity (Figure 1) [14,15]. Immunity mechanisms can involve a single small protein, a complex of proteins, a membrane-bound metalloprotease, multi-drug transporter proteins, or the combined action of a cognate immunity protein and ABC transporter [16].

**Figure 2:**
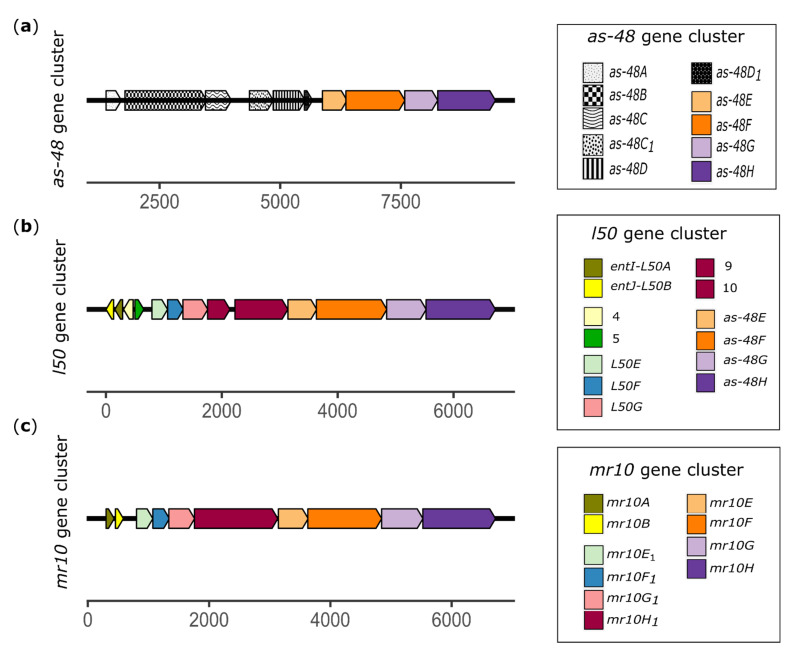
Genetic organization of AS-48, MR10A/B and L50A/B enterocins. (**a**) Ten identified open reading frames (ORFs) are depicted in the *as-48* gene cluster, all running in the same direction: *as-48A* (dotted pattern) is the structural gene; *as-48B* (squared pattern) appears to be involved in biosynthetic machinery; *as-48C* (wavy pattern) may encode an auxiliary immunity protein; *as-48C*_1_ (large dotted pattern) and *as-48D* (striped pattern) form the first pump involved in bacteriocin secretion; *as-48D*_1_ (black) encodes an immunity protein; *as-48E* (light orange), *as-48F* (dark orange), *as-48G* (light violet), and *as-48H* (dark violet) form a second pump constituted by an ABC transporter-2 involved in self-immunity. (**b**) Gene cluster of L50A/B enterocins. A total of 13 identified ORFs [26] are depicted. *l50A* and *l50B* are the structural genes for enterocins (yellow and olive-green arrows), which are arranged in the opposite orientation to the remaining genes. *orf4* and *orf5* (beige and green arrows) with unknown functions. *L50E*, *L50F*, and *L50G* (light blue, blue and pink arrows, respectively) may encode transporters of the ABC type *orf9* and *orf10* (maroon arrows) with unknown function. Finally, *as-48E* (light orange), *as-48F* (dark orange), *as-48G* (light violet), and *as-48H* (dark violet) constitute a second pump formed by an ABC transporter-2. (**c**) Gene cluster of MR10A/B enterocins. Ten identified ORFs are depicted, all running in the same direction. *mr10A* and *mr10B* (olive green and yellow arrows) are the structural genes for MR10A and MR10B enterocins; *mr10E*_1_ (light blue arrow) encodes a DUF (domain of unknown function) protein family; *mr10F*_1_ and *mr10G*_1_ (blue and pink arrows) encode two proteins of unknown function. *mr10H*_1_ gene (maroon arrow) encodes a PH domain-containing protein, and *mr10E*, *mr10F*, *mr10G*, and *mr10H* (light orange, dark orange, light violet, and dark violet arrows, respectively) represent a pump constituted by an ABC transporter.

One of the best-characterized enterocinogenic systems is the cyclic peptide AS-48 (Class I) produced by *Enterococcus faecalis* [17]. The *as-48* gene cluster (Figure 2a) is located in the conjugative plasmid pMB2 [18] and comprises at least ten genes (*as-48A*, *B*, *C*, *C*_1_, *D*, *D*_1_, *E*, *F*, *G* and *H*); their transcriptional analysis revealed two polycistronic mRNAs that correspond to the expression of *as-48ABC* and *as-48C*_1_*DD*_1_*EFGH* operons, respectively [17]. This analysis also identified an internal P_D1_ promoter involved in the transcription of *as-48D*_1_*EFGH* genes [19]. The structural gene *as-48A* encodes a protein of 105 amino acids, of which the first 35 constitute a signal peptide preceded by a strong promoter (P_A_) that also directs the expression of *as-48BC* genes, which are separated from the structural gene by a short but important inverted repeat (IR) [17,19]. The *as-48B* gene encodes a protein that may participate in the biosynthetic machinery, *as-48C* appears to encode an accessory immunity protein, and *as-48D*_1_ encodes an immunity determinant protein [20]. This cluster also contains two ABC transporters: *as-48C*_1_*D* (Transporter-1), responsible for secretion of the enterocin, and *as-48EFGH* (Transporter-2), which provides additional self-protection against the enterocin [17]. This type of Transporter-2 has been described in bacteriocins that produce pores in the cytoplasmic membrane, removing the bacteriocin from producer cells and keeping the bacteriocin concentration in the cytoplasmic membrane below the critical level necessary for pore formation [21].

The *as-48EFGH* genes comprise an ABC transport system observed in a different class of bacteriocins such as enterocins L50A/B. These bacteriocins (L50A/B) are frequent among enterococci [22,23,24,25], and several variants have been identified, including EntJ/I [26], F58 [27], and MR10A/B [28]. Ruiz-Barba et al. [26] sequenced the plasmid pEF1 from *Enterococcus faecium* 6T1, which is responsible for ENT I/J production. In addition to the structural genes *ent I/J*, they described three additional downstream genes of unknown function that are virtually identical to *L50 EFG* and a set of genes similar to the AS-48EFGH ABC transport system in the *as-48* gene cluster (Figure 2b).

Bacteria possess mechanisms to protect themselves from their own bacteriocins; for most bacteriocins, however, the mechanisms involved in immunity remain poorly understood [16]. Our group has observed cross-resistance between enterococcal strains carrying the *as-48* gene cluster, and other bacteriocins such as MR10A/B (unpublished data). Cross-resistance arises in various situations: when the two cells have a common receptor for the antimicrobial substance, when the antimicrobial agent initiates a common pathway to cell death, or when they share a common route of access to their respective targets [29]. Evidence is emerging of cross-resistance between bacteriocins [30,31,32], although cross-resistance has only been observed between closely related bacteriocins to date [33,34], and nothing is known about cross-resistance between different classes of bacteriocins. Consequently, the aim of the present study was to elucidate the mechanisms underlying cross-resistance between different classes of antimicrobial peptides based on the AS-48 Transporter-2 system. The study hypothesis was that the ABC Transporter-2 system in the *as-48* gene cluster could be responsible for cross-resistance with other classes of bacteriocins, bestowing this system with resistance beyond its own antimicrobial peptide.

This study was designed to elucidate the role of the AS-48EFGH ABC transporter in cross-resistance to the bacteriocins MR10A/B by analyzing the sensitivity to this bacteriocin of a collection of different mutants of the AS-48 bacteriocin gene cluster. In addition, analysis of the *mr10A/B* gene cluster revealed a degree of similarity between AS-48 Transporter-2 and MR10A/B ABC transporter.

## 2. Materials and Methods

### 2.1. Bacterial Strains and Culture Media

The bacterial collection used in the study encompasses 21 strains (Table 1). *E. faecalis* MRR 10-3, A-48-32, and *E. faecium* F58 were grown on Trypticase Soy Agar (TSA) at 37 ºC. *E. faecalis* strains JH2-2 (pAM401) and JH2-2 (pAM401-81), and the set of Tn*5* mutants were cultured in Trypticase Soy Broth (TSB) or TSA with chloramphenicol (20 µg/mL) to avoid plasmid curing during their growth.

Brain Heart Infusion Agar (BHA) and Mueller–Hinton Agar (MHA) were used for inhibition assays, buffering the media in 0.1 M sodium phosphate buffer at pH 6.9 to avoid interference from any inhibitory effect of organic acids produced in their fermentative metabolism.

Mutant D1Pst1 was constructed by *Pst* I digestion of plasmid 401-81::Tn5_D_. A fragment of 14 kb was purified, religated, and used to transform *E. faecalis* JH2-2, as previously described [20].

### 2.2. Inhibitory-Activity Assays

The agar well diffusion method was used to follow the antimicrobial substance during the purification process [39,40]. Briefly, stainless steel cylinders with outer diameter of 8 mm were placed on the agar plate surface and then overlayed with 6 mL of soft BHA inoculated with 2% of the indicator strain culture. After this overlay solidified, cylinders were removed, and 70 µL of the solution to be tested was introduced into the well.

The drop-plating technique was also used to determine the sensitivity of the different mutants to the purified bacteriocin. Briefly, an overlay of soft BHA inoculated with a 2% indicator strain was poured onto the surface of the BHA plate. After this overlay solidified, 5 µL drops of the purified bacteriocin, two-fold diluted up to 1/16, were placed onto the plates. Inhibition halo diameters were measured after 24 h of incubation at 37 °C.

### 2.3. Bacteriocin Production and Purification

Enterocins MR10A/B were purified from 1 L of buffered BHI inoculated with an overnight culture of MRR 10-3 strain and incubated at 37 °C for 10 h. Bacteriocins were recovered by cation-exchange chromatography on carboxymethyl-Sephadex CM-25 (Amersham). Active fractions were identified using *Listeria innocua* as indicator strain and concentrated through reversed-phase chromatography by hydrophobic interaction with a C18 column (Waters Corporation, Milford, MA, USA) [28]. Finally, active fractions from the column were lyophilized, dissolved in 1.5 mL of 0.05% acetic acid, and stored at −20 °C. By this procedure, the bacteriocin was concentrated up to 600× with respect to the initial culture concentration.

### 2.4. mr10A/B Gene Cluster Sequencing and Annotation, and Genetic Data

The *mr10A/B* gene cluster (accession no. MW689545) was obtained from the partial sequencing of the *E. faecalis* MRR10-3 genome. The genome library was constructed using a TruSeq DNA PCR-free library preparation kit (Illumina, Inc., San Diego, CA, USA) with an insert size of 350 bp sequenced at Macrogen, Inc. (Seoul, Republic of Korea) with a HiSeq Illumina platform by paired-end sequencing of 2 × 101 bp read lengths. The genomes were assembled with SPAdes 3.13 [41] and annotated with Prokka 1.13.3 [42].

When necessary, the function of *mr10A/B* cluster genes was assigned by searching for homologies with the protein sequence using BLASTP (version 2.11.0+) on an NCBI server with a non-redundant database.

Homologies between *mr10A/B*, *l50*, and *as-48* gene clusters were revealed by aligning in pairs using Blastn suit-2 sequences [43].

Comparisons between MR10A/B and AS-48 pump-forming proteins and between *mr10A/B* and *l50* gene cluster proteins were performed by comparing pairs using Blastp suit-2 sequences [44,45].

### 2.5. Cluster Analyses

Mutants were grouped according to their sensitivity to MR10A/B by performing K-means clustering in R (version 3.6.3) [46] using Rstudio (version 1.1.447) [47]. First, K-means functioning was used to calculate the optimal number of clusters by the Elbow method. Next, the sensitivity of each group of mutants was exhibited in a heatmap, constructing a dendrogram by the complete-linkage method with ComplexHeatmap [48], viridis [49], Stats [46], and dendextend [50] packages.

## 3. Results

### 3.1. mr10A/B Gene Cluster

The *mr10A/B* gene cluster is formed by at least 10 genes (*mr10A*, *mr10B*, *mr10E1*, *mr10F1*, *mr10G1*, *mr10H1*, *as48E*, *as48F*, *as48G*, *mr10H*) (Figure 2c). Structural genes *mr10A* and *mr10B* encode two leaderless proteins of 134 and 131 amino acid residues, respectively. The putative gene *mr10E1* encodes a Domain of Unknown Function (DUF) protein family. The putative genes *mr10F1* and *mr10G1* also encode two proteins of unknown function (90 and 141 aa, respectively). The putative *mr10H1* gene encodes a Pleckstrin Homology (PH) domain-containing protein (458 aa). Additionally, the *mr10EFGH* gene cluster constitutes an ABC transport system in which *mr10E* encodes a protein of 163 amino acid residues of unknown function, *mr10F* (406 aa) encodes an efflux Resistance-Nodulation-Division (RND) transporter periplasmic adaptor subunit, *mr10G* (227 aa) encodes an ATP-binding protein, and finally, *mr10H* encodes the ABC transporter permease.

### 3.2. Homologies between the ABC Transporters

Given the presence of the AS-48 ABC Transporter-2 in the *mr10A/B* gene cluster, the DNA coding sequences for AS-48 (accession no. Y12234 and AJ438950) and MR10A/B genes were compared to quantify their similarity. Blast alignment showed 95.978% identity in the DNA region that includes the *as-48EFGH* genes. Individually, each protein of the ABC transporter showed a high similarity (>95%) except for As-48E and Mr10E, which evidenced 46% identity (Table 2).

**Figure 3:**
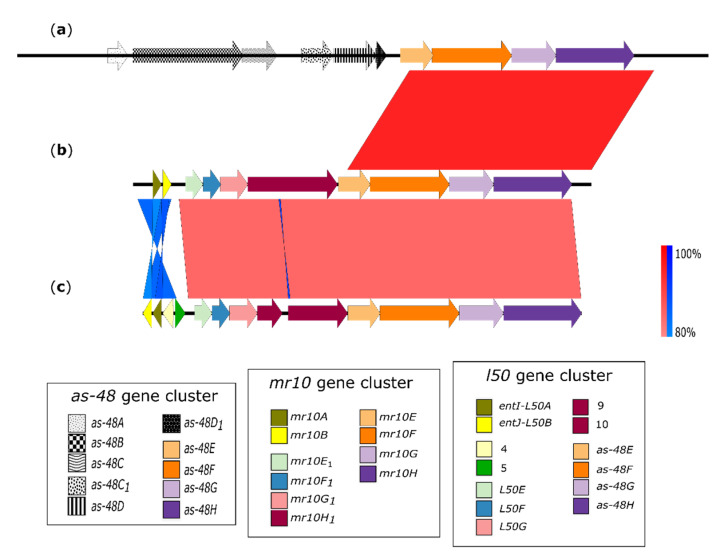
Homology between gene clusters of enterocins AS-48 (**a**), MR10A/B (**b**), and L50A/B (**c**). The color scale represents the similarity between the different genes: light red and blue = 80% similarity, dark red and blue = up to 100% similarity; red corresponds to direct similarity and blue to inverse similarity. Homologies between *mr10A/B* and *as-48* clusters appear in the last four genes corresponding to ABC Transporter-2.

In addition, the two gene clusters were compared to analyze differences between the homologous bacteriocins MR10A/B and L50A/B [26]. Both gene clusters had 83% identity at DNA level, although there was an inversion in the structural genes (Figure 3). High similarities (around 70–87%) were obtained in comparisons of the protein sequence of each individual gene (Table S1). However, the *l50* gene cluster described by Ruiz-Barba et al. [26] (accession no. DQ198088.1) contains some additional genes. Nevertheless, the *l50* gene cluster re-annotated in RefSeq (accession no. NC_010880.1) matched our annotation of the *mr10A/B* gene cluster, showing the same percentage identity as previously obtained.

### 3.3. Mutant Sensitivity to Enterocins MR10A/B

**Figure 4:**
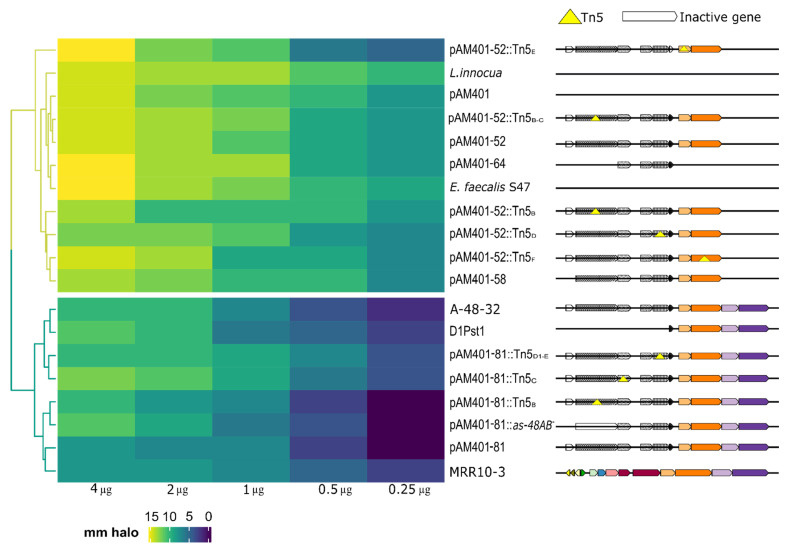
Sensitivity of AS-48 mutants to MR10A/B. Heatmap depicting the cluster analysis of mutant sensitivity against MR10A/B. Two main clusters emerge: sensitive strains (yellow branches) and resistant strains (blue branches). The sensitive cluster includes all strains without a complete functional ABC Transporter-2, whereas the resistant cluster includes all strains with a complete functional ABC transporter-2. The dendrogram shows the sensitivity of the mutant strains (rows) to different concentrations of MR10A/B (columns). The color scale represents the millimeters of halo around the colony, from dark blue (absence of halo) to yellow (largest halo (15 mm)). The righthand side shows the gene cluster for each mutant: *as-48A*, dotted arrow; *as-48B*, squared arrow; *as-48C*, wavy arrow; *as-48C*_,_ large, dotted arrow; *as-48D*, striped arrow; *as-48D*, black arrow; *as-48E*, light orange arrow; *as-48F*, dark orange arrow; *as-48G*, light violet arrow; and *as-48H*, dark violet arrow. We used 401-81; MRR10-3 and A48-32 (wild phenotypes) served as positive controls and *L. innocua*, *E. faecalis* S-47, and *E. faecalis* 401 as negative controls (non-producers of AS-48 or MR10A/B). *Tn5* is represented by a yellow triangle and an inactivated gene by a white arrow.

After MR10A/B bacteriocin purification and concentration, inhibitory assays were performed using several mutants of *as-48* gene cluster as indicator strains. The inhibition halo around the colony was measured to determine the degree of sensitivity of each strain (Figure 4 and Figure S1 and Table S2).

Analysis of the sensitivity of AS-48 mutants to MR10A/B clusters yielded two well-differentiated phenotypic clusters: resistant and sensitive. The common characteristic of resistant mutants was that *as-48EFGH* genes, which encode ABC transporter-2, were intact. Resistance persisted even when other genes of cluster *as-48* were interrupted by Tn5. In fact, the resistance to bacteriocin was even maintained when all genes in the cluster were deleted except for *as-48D*_1_*EFGH*, as in the mutant D1Pst1 (Figure 4). Conversely, all sensitive strains evidenced a deletion of one or more *as-48EFGH* genes. The main deletions involved *as-48G* and *as-48H* genes.

## 4. Discussion

Antimicrobial assays of MR10A/B enterocins against a mutant collection of the *as-48* gene cluster showed that ABC transporters play a role in cross-resistance between bacteriocins of different classes. Although defined as antimicrobial peptides active against close relatives, a wide range of bacteriocins possess activity against more distantly related bacteria [6]. In addition to the role of the resistance genes in self-immunity, the present results show that they can act against several types of antimicrobials and may therefore produce different degrees of resistance to both closely and distantly related bacteria.

Enterocins MR10A/B are variants of L50A/B [28], i.e., class II bacteriocins. A previous study only characterized the structural genes of MR10A/B [28], whereas the present investigation reveals the genetic composition of the whole *mr10A/B* gene cluster, which contains the following 10 genes: two structural genes *(mr10A* and *mr10B*), corresponding to MR10A and MR10B enterocins; four genes (*mr10E1*, *mr10F1*, *mr10G1*, and *mr10H1*) with unknown function and four genes (*mr10EFGH*) that form an ABC transport system. Comparison between *mr10A/B* and the *l50* gene clusters revealed the presence of two additional genes (*orf4*, *orf5*). In addition, *mr10H1* appears split into the open reading frames (ORFs) *orf9* and *orf10* [26] (Figure 3), and each protein of these two ORFs has a similarity greater than 70% with our annotated protein Mr10H1. Nevertheless, our annotation fits the re-annotation of the *l50* gene cluster included in RefSeq of the NCBI (Figure 3).

Bacteriocins constitute an active mechanism used by bacteria to antagonize competitors and promote their own survival [51]. Bacteriocins produced by LAB are important natural food preservatives and also act against bacterial pathogens, representing a viable alternative to antibiotics [52]. Several studies have demonstrated the possibility of cross-resistance between bacteriocins produced by closely related bacterial strains. Fimland et al. [33] studied curvacin A, enterocin A and enterocin B, enterocin P, leucocins A and C, pediocin PA-1 and Sakacin P, which are classified as class II bacteriocins, and reported that a strain transformed with the specific immunity gene for one bacteriocin could show resistance to others in the same class. Oppengård et al. [34] studied the two-peptide bacteriocins lactococcin G and enterocin 1071, which are homologous and belong to the same Class (Class II); they found that *Lactococcus* sp. transformed with the enterocin 1071 immunity gene are protected against both enterocin 1071 and lactococcin G, whereas lactococci transformed with the lactococcin G immunity gene were not protected against enterocin 1071. Neither of these immunity proteins protected the lactococci against the two-peptide bacteriocin plantaricin EF, a bacteriocin which is not homologous to lactococcin G or enterocin 1071 [34].

In this study, cross-resistance was observed between MR10A/B and AS-48 bacteriocins, which belong to different classes (Class II and I, respectively). Antimicrobial activity assays of our *as-48* mutant collection against MR10A/B revealed the functional role of ABC Transporter-2 (*as-48EFGH*) in this phenomenon. It was found that resistance was retained by mutants with a complete ABC transporter but not by those with absent or incomplete ABC transporter, which were sensitive to the enterocin. This finding suggests that the cross-resistance mechanism of AS-48 and MR10A/B producer strains is based on the presence of functional efflux pumps (ABC transport). The presence of these pumps in the bacterial membrane help to expel antibacterial substances before they reach their target, thereby providing immunity [53,54]. Furthermore, in addition to its own specific immunity protein, the ABC Transporter-2 was found to provide the producer with resistance to other enterocins, even to those in a different bacteriocin class. Another explanation may be that AS-48 and MR10A/B share the following structural characteristics: (i) a three-dimensional structure similar to a saposin-like fold or α-helical bundle [55]; (ii) a hydrophobic core formed by the helices (typically 4 or 5), with an outer surface that is predominantly hydrophobic and has solvent-exposed tryptophan or tyrosine residues close to the N or C-termini [55]. These characteristics may favor the recognition by ABC Transporter-2 of both peptides and their expulsion to the extracellular medium, conferring resistance. Nevertheless, we observed that the ABC transport present in the *mr10A/B* gene cluster confers lesser resistance to enterocin AS-48 (data not shown).

The long misuse of antibiotics has increased the number of multidrug-resistant pathogens [56], generating a high-priority health problem [57]. Various alternatives to classic antibiotics are currently under study, including antimicrobial peptides of microbial origin (e.g., bacteriocins) or those from host cells [58]. However, it is critical to examine the mechanisms and dynamics of resistance associated with an alternative approach. Unlike traditional antibiotics, antimicrobial peptides can interact with the microbial membrane by neutralizing the membrane charge and/or enter the cytoplasm of the cell, producing bacterial death [58]. They can kill germs rapidly at low concentrations and have even proven effective against antibiotic-resistant strains [58]. Their mechanisms of action involve multiple low-affinity targets rather than a single high-affinity target, which is the objective of antibiotics [59]. For this reason, bacteriocins have been considered at low risk of developing resistance. However, in vivo and in vitro studies have reported that bacteria exposed to therapeutic antimicrobial peptides can select antimicrobial peptide-resistant strains [60,61,62]. It is therefore crucial to determine potential patterns of cross-resistance in order to predict cross-resistance between bacteriocins. This knowledge can also help to prevent bacterial cross-resistance to the microbicidal action of human antimicrobial peptides, on which the innate immune system depends. These results also have major implications for the modeling of production/resistance patterns in wild populations and open the way to investigating this cross-resistance in other antimicrobial peptide systems.

## Figures and Tables

**Table 1:** Bacterial strains used in the present study.

Strain **	Mutation	Reference or Source ^a^
JH2-2 (pAM401-81)	*as-48ABCC*_1_*DD*_1_*EFGH* gene cluster cloned in pAM401 vector	[21]
D1Pst1 *(as-48DEFGH* cloned from 401-81::Tn5_D1_ mutant)	*as-48D*_1_ *EFGH*	This study
JH2-2 (pAM401-81::Tn*5*_D1-E_)	*as-48ABCC*_1_ *DD*_1_ *EFGH	[21]
JH2-2 (pAM401-81::Tn*5*_C_)	as-48ABC*C_1_ *DD*_1_ *EFGH*	[35]
JH2-2 (pAM401-81::Tn*5*_B_)	as-48AB*CC_1_ *DD*_1_ *EFGH*	[35]
JH2-2 (pAM401-81::*as-48AB^-^*)	*as-48CC*_1_ *DD*_1_ *EFGH*	[21]
JH2-2 (pAM401-52)	*as-48ABCC*_1_ *DD*_1_ *EF*	[20]
JH2-2 (pAM401-52::Tn*5*_F_)	*as-48ABCC*_1_ *DD*_1_ EF*	[20]
JH2-2 (pAM401-52:: Tn*5*_E_)	*as-48ABCC*_1_ *DD*_1_ E*F	[20]
JH2-2 (pAM401-52::Tn*5*_D_)	*as-48ABCC*_1_ D*D_1_ *EF*	[20]
JH2-2 (pAM401-52::Tn*5*_B-C_)	as-48AB*CC_1_ *DD*_1_ *EF*	[20]
JH2-2 (pAM401-52::Tn*5_B_*)	as-48AB*CC_1_ *DD*_1_ *EF*	[20]
pAM401-58 (pAM401-52::*as48A^-^*)	*as-48BCC*_1_ *DD*_1_ *EF*	[35]
pAM401-64	ABC transporter cloned into pAM401 *as-48CC*_1_*DD*_1_	[20]
*E. faecalis* MRR10-3	Wild type	[28]
*E. faecium* F58	Wild type	[36]
*E. faecalis* A-48-32	Wild type AS-48 producer	[18]
JH2-2 (pAM401)	Negative control	[37]
*L.innocua* 4030	Wild type Indicator strain	CECT
*E.faecalis* S-47	Wild type Indicator strain	[38]

(*) Tn5 inserted in gene. (**) All species correspond to *E. faecalis* unless otherwise indicated. ^a^ CECT, Spanish Type Culture Collection.

**Table 2:** Comparison between MR10A/B and AS-48 pump-forming proteins. The table represents the similarity observed between the ABC transporter proteins of MR10A/B and AS-48.

As-48EFGH	Percentage Identity (%)	MR10EFGH
As-48E	46.01	Mr10E
As-48F	97.74	Mr10F
As-48G	99.56	Mr10G
As-48H	95.55	Mr10H

## Data Availability

Sequences are available at NCBI nucleotide under accession numbers: MW689545 (*mr10* gene cluster), Y12234 and AJ438950 (*as-48* gene cluster), DQ198088.1 (*l50* gene cluster, GenBank), NC_010880.1 (*l50* gene cluster, RefSeq).

## Supplementary Materials

The following are available online at https://www.mdpi.com/article/10.3390/microorganisms9071411/s1, Table S1: Comparison between putative proteins of *mr10* and *l50* gene clusters, Table S2: Inhibition halo (expressed in mm) of each *E. faecalis* strain against different concentrations of MR10A/B bacteriocin, Figure S1: Antibacterial activity assays.
