# Supplementary material for: Enterocin Cross-Resistance Mediated by ABC Transport Systems
Source: Microorganisms. 2021 Jun 30;9(7):1411. doi: 10.3390/microorganisms9071411 (PMC8306556; doi:10.3390/microorganisms9071411)
Supplement: Supplementary file 1 [file microorganisms-09-01411-s001.zip › microorganisms-1238555-supplementary.pdf]

Table S1. Comparison between putative proteins of *mr10* and *l50* gene clusters. Table shows similarity between the proteins of the *mr10* cluster (MW689545) and the *l50* cluster deposited in Refseq (accession no. NC_010880.1) and in Genbank (accession no. DQ198088.1).

***l50* cluster Refseq**	**Per.Ident (%)**	***mr10* cluster**	**Per.Ident (%)**	***l50* cluster Genbank**
L50E	87.64%	Mr10E1	87.64%	L50E
Orf7	74.44%	Mr10F1	74.44%	L50F
Orf8	79.02%	Mr10G1	79.02%	L50G
Orf9	77.60%	Mr10H1	77.60%	Orf9
-	-	Mr10H1	74.09%	Orf10
As-48E	73,62%	Mr10E	73,62%	As-48E
As-48F	75.68%	Mr10F	75.68%	As-48F
As-48G	92.51%	Mr10G	92.51%	As-48G
As-48H	89.22%	Mr10H	89.22%	As-48H

Table S2. Inhibition halo (expressed in mm) of each *E. faecalis* strain against different concentrations of MR10A/B bacteriocin.

	**1 (4 μg)**	**2 (2 μg)**	**3 (1 μg)**	**4 (0.5 μg)**	**5 (0.25 μg)**
**(a) JH2-2 (pAM401)**	14	12	11	10	8
**(b) pAM401-64**	15	13	13	9	8
**(c) JH2-2(pAM401-52)**	14	13	11	9	8
**(d) JH2-2(pAM401-52::Tn5$_F$)**	14	13	9	9	7
**(e) JH2-2(pAM401-52::Tn5$_{B-C}$)**	14	13	12	9	8
**(f) JH2-2(pAM401-52::Tn5$_D$)**	12	12	11	8	7
**(g) *E.faecalis* MRR10-3**	8	8	7	5	3
**(h) *E. faecalis* A-48-32**	10	10	7	4	2
**(i) JH2-2(pAM401-81)**	8	7	6	3	0
**(j) D1Pst1**	11	10	6	5	3
**(k) JH2-2 (pAM401-81:: *as-48AB$^-$*)**	11	9	6	4	0
**(l) JH2-2(pAM401-81::Tn5$_B$)**	10	8	7	3	0

(I)

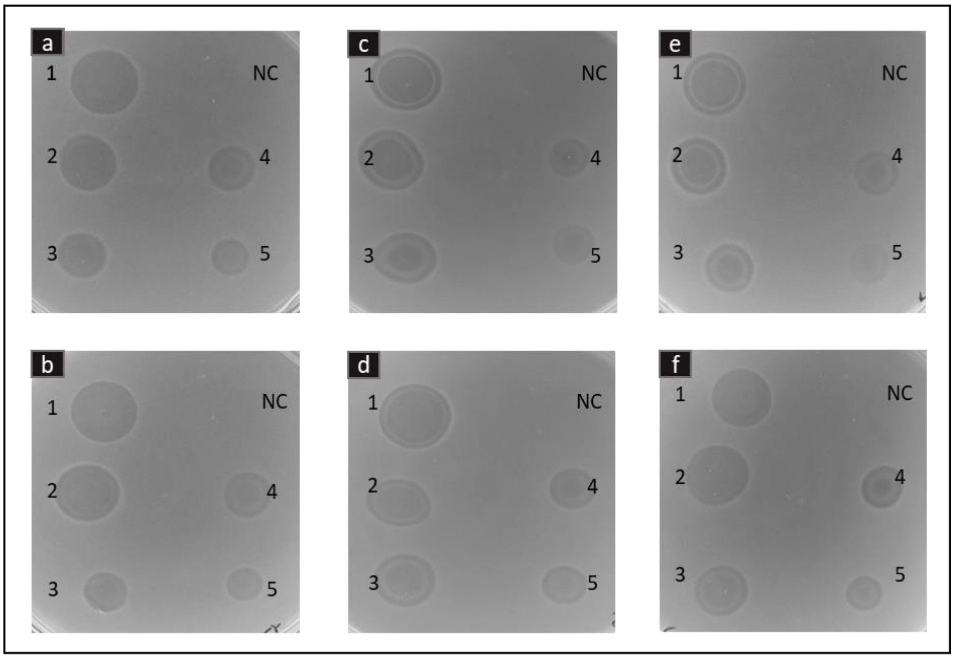

(II)

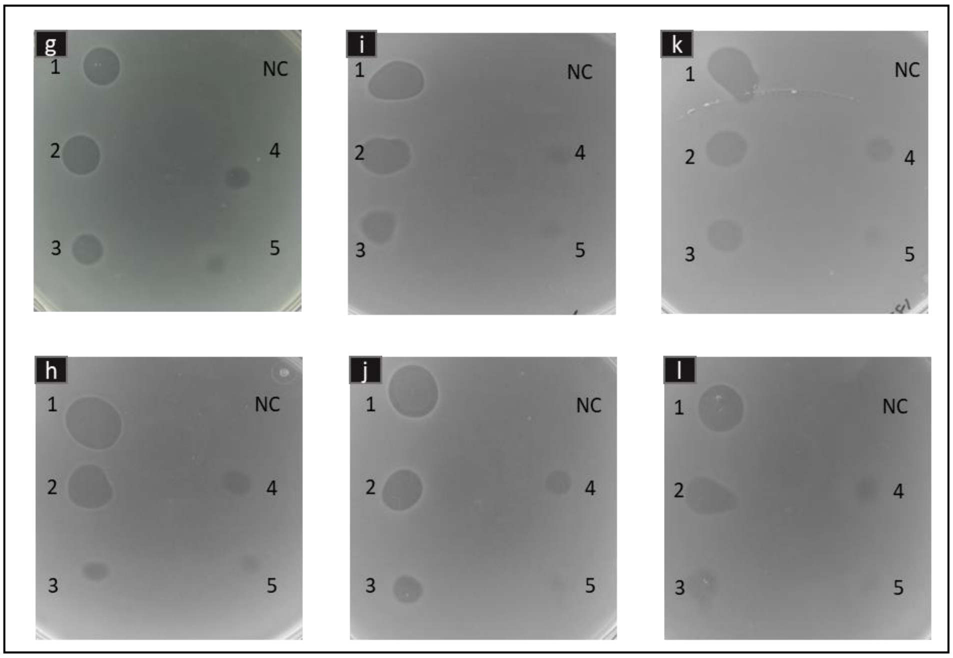

Figure S1.Antibacterial activity assays. (I) *E. faecalis* AS-48 strains without a functional AS-48 Transporter-2 against different concentrations of MR10A/B bacteriocin. (II) *E. faecalis* AS-48 strains with a functional AS-48 Transporter-2 against different concentrations of MR10A/B bacteriocin. Letters (a) to (l) represent each mutant: (a) JH2-2(pAM401), (b) JH2-2(pAM401-52::Tn5$_{F}$), (c) pAM401-64, (d) JH2-2(pAM401-52::Tn5$_{B-C}$), (e) JH2-2(pAM401-52), (f) JH2-2(pAM401-52::Tn5$_{D}$), (g) *E.faecalis* MRR10-3, (h) D1Pst1, (i) *E. faecalis* A-48-32, (j) JH2-2(pAM401-81::as-48AB^{-}), (k) JH2-2(pAM401-81) and (l) JH2-2(pAM401-81::Tn5$_{B}$). Numbers 1 to 5 represent amount of bacteriocin available in each spot, where: (1) 4 µg, (2) 2 µg, (3) 1 µg, (4) 0.5 µg and (5) 0.25 µg. (NC) Negative control (Bacteriocin dilution solution (acetic acid 0.05 %)). The size of each inhibition halo are shown in **Table S2.**
